# Special Issue “Anaerobes in Biogeochemical Cycles”

**DOI:** 10.3390/microorganisms9010023

**Published:** 2020-12-23

**Authors:** Caroline M. Plugge, Diana Z. Sousa

**Affiliations:** Laboratory of Microbiology, Wageningen University & Research, Stippeneng 4, 6708 WE Wageningen, The Netherlands; diana.sousa@wur.nl

Anaerobic microorganisms, Bacteria and Archaea, have an essential role in global biogeochemical cycles. Anaerobes are redox specialists and are responsible for the natural recycling of redox-active chemical elements which are abundant in the biosphere (carbon, nitrogen, sulphur, iron, and phosphorus) as well as of various other elements present in small amounts, such as manganese [1,2,3]. Biogeochemical cycles influence our climate, wastewater treatment, biofuels production, are essential for food production, and contribute to important processes in our intestinal tract.

The anaerobic cycling of nutrients requires complex microbiome interactions. An exceptionally diverse world of microorganisms inhabits the anaerobic environments on earth. These microorganisms obtain their energy by fermentation and anaerobic respiration; in addition, some phototrophic and chemoautotrophic processes operate in the absence of oxygen. Despite the fundamental importance of anaerobes, many uncertainties remain about their diversity and physiology and the processes in which they are involved [1].

This Special Issue gathers seven articles on anaerobes with roles in several biogeochemical cycles. One article involves the inorganic carbon cycle [4], two describe the relationship between predominant physiological types of prokaryotes in marine sediments and propionate and butyrate degradation through sulphate reduction, fermentation, and methanogenesis in marine sediments [5,6], two involve the discovery of novel biodiversity involved in the sulphur cycle [7,8], and two focus on the role of iron in anaerobic methane oxidation [9] and in the facilitation of anaerobic long chain fatty acid degradation [10].
